# Salvianolate Protects Hepatocytes from Oxidative Stress by Attenuating Mitochondrial Injury

**DOI:** 10.1155/2016/5408705

**Published:** 2016-06-02

**Authors:** Qiang Zhao, Yuan Peng, Kai Huang, Yang Lei, Hong-Liang Liu, Yan-Yan Tao, Cheng-Hai Liu

**Affiliations:** ^1^Institute of Liver Diseases, Shuguang Hospital Affiliated to Shanghai University of Traditional Chinese Medicine, 528 Zhangheng Road, Shanghai 201203, China; ^2^Shanghai Key Laboratory of Traditional Chinese Clinical Medicine, 528 Zhangheng Road, Shanghai 201203, China; ^3^E-Institute of TCM Internal Medicine, Shanghai Municipal Education Commission, 1200 Cailun Road, Shanghai 201203, China

## Abstract

Salvianolate is widely used to treat angiocardiopathy in clinic in China, but its application in liver diseases remains unclear. Our study aims to investigate the effect of Salvianolate on rat hepatic injury by protecting hepatocyte mitochondria. To evaluate the effects of Salvianolate on injured hepatocytes, alpha mouse liver 12 (AML-12) cells were induced with hydrogen peroxide (H_2_O_2_) and treated with Salvianolate. Cell viability and MitoTracker Green for mitochondria and 5,5′,6,6′-tetrachloro-1,1′,3,3′-tetraethylbenzimidazole-carbocyanide iodine (JC-1) levels and cytochrome C (Cyto-C) expressions were detected* in vitro*. To identify the effect of Salvianolate on protecting against mitochondria injury, male Wistar rats were injected with carbon tetrachloride (CCl_4_) and treated with Salvianolate (40 mg·kg^−1^). Serum liver function, parameters for peroxidative damage, hematoxylin and eosin (H&E) staining, and transmission electron microscope (TEM) of hepatocyte mitochondria were assayed. Our results showed that Salvianolate effectively protected hepatocytes, increased mitochondria vitality, and decreased Cyto-C expressions* in vitro*. Besides, Salvianolate alleviated the liver function, attenuated the indicators of peroxidation, and relieved the mitochondria injury* in vivo*. In conclusion,* Salvianolate* is effective in protecting hepatocytes from injury* in vitro* and* in vivo*, and the mechanism might be related to its protective effect on hepatocyte mitochondria against oxidative stress.

## 1. Introduction

Hepatocyte injury could be caused by a wide range of risk factors, including viruses, drugs, metabolic disorders, and immune attacks [1]. When hepatocytes are damaged, a large number of cytokines will be released, which further aggravates liver injury and eventually leads to liver fibrosis. Thus, protecting hepatocytes is of vital importance for curing varieties of acute and chronic liver diseases. And searching for an appropriate drug with strong protective effect against hepatocyte injury is a key point in healing live diseases.

Mitochondria are the main organelle producing alanine aminotransferase (ATP) and providing majority of the energy for cells physiological needs [2]. Moreover, mitochondria also play important roles in intracellular signal transduction and differentiation and apoptosis of cells [3–5]. Oxidative stress, related to mitochondria injury, is a common pathological mechanism for hepatocyte injury caused by etiologic agents [6, 7]. Therefore, antagonizing mitochondrial damage could effectively protect hepatocytes from injury [8–10].

Salvianolate, the main water soluble compound extracted from Radix* Salvia miltiorrhiza*, is widely used in clinic in treating cardiovascular diseases by inhibiting reactive oxygen species (ROS) production [11]. However, its pharmacological effect on liver diseases remains unclear.

In this study, we aimed to observe the effects of Salvianolate on hepatocyte mitochondria injury. To clarify our aim, several questions should be formulated. Specifically, they are as follows. (1) Could Salvianolate inhibit hepatic injury? (2) If so, could Salvianolate protect hepatocytes by inhibiting mitochondria injury? Our results demonstrated that Salvianolate could reduce hepatocyte mitochondria injury. These findings might suggest a cytoprotective therapeutic potential of Salvianolate in the context of mitochondria injury-mediated liver diseases.

## 2. Materials and Methods

### 2.1. Drug

Salvianolate (Lot. number 120418) was provided by Shanghai Green Valley Pharmaceutical Co., Ltd.; N-acetyl-L-cysteine (NAC) (Lot. number WXBB2161V) was purchased from Sigma Chemical Co., Ltd.

### 2.2. Reagents

CCl_4_ and H_2_O_2_ were obtained from Sinopharm Group Co., Ltd. All the other reagents were of the highest grade commercially available.

### 2.3. Cell Culture

Alpha mouse liver 12 (AML-12) cell line was purchased from Shanghai Life Science Research Institute, Chinese Academy of Sciences Institute of Biochemistry and Cell Biology. AML-12 cells were cultured in a 1 : 1 mixture of Dulbecco's Modified Eagle's Medium (DMEM) and Ham's F12 medium supplemented with 0.005 mg/mL insulin, 0.005 mg/mL transferrin, 5 ng/mL selenium, 40 ng/mL dexamethasone, 10% fetal bovine serum (FBS), 100 units/mL of penicillin, and 100 units/mL of streptomycin. All the cells were grown in a humidified incubator at 37°C in a 5% CO_2_ atmosphere.

### 2.4. Drug Treatments

Salvianolate was initially dissolved as concentrated stock solution in 1 mg/mL sterile phosphate-buffered saline (PBS). For evaluating the effect of Salvianolate on AML-12 cells* in vitro*, AML-12 cells were cultured in a 96-well plate (Corning, NY, USA) at a density of 5,000 cells/well and were incubated with Salvianolate at the concentration of 3.125–400 *μ*g/mL for 24 hours.

### 2.5. Cell Viability Assay

After the cells were incubated with Salvianolate at the concentration of 3.125–400 *μ*g/mL for 24 hours, the cell viability assays were performed using the Cell Counting Kit 8 (CCK8) (Beyotime Biotechnology, Jiangsu, China), according to the manufacturer's protocol.

### 2.6. MitoTracker Staining

For live cell analysis, AML-12 cells were cultured in a 96-well plate at a density of 5,000 cells/well and were incubated with Salvianolate at the concentration of 6.25 *μ*g/mL, 12.5 *μ*g/mL, and 25 *μ*g/mL for a period of 24 hours. Before the experiment, the cells were stained with 50 nM MitoTracker Green (Beyotime, Jiangsu, China) for mitochondria and then were treated as the manufacturer's protocol in the cellular retention assay. Data were collected by using fluorescence microplate reader (Molecular Devices, Inc., Sunnyvale, California, USA). The fluorescence of MitoTracker Green (green) was excited and collected at 473/516 nm.

### 2.7. JC-1 Staining

AML-12 cells were cultured in a 96-well plate at a density of 5,000 cells/well and were incubated with Salvianolate at the concentration of 6.25 *μ*g/mL, 12.5 *μ*g/mL, and 25 *μ*g/mL for a period of 24 hours. Then mitochondrial membrane potential (MMP) was assessed by JC-1 (Beyotime, Haimen, China) staining. Briefly, AML-12 cells were stained with JC-1 for 15 min at 37°C. Cells treated with 10 *μ*M carbonyl cyanide m-chlorophenylhydrazone (CCCP) were used as negative control. CCCP is a protonophore which can cause dissipation of MMP. Cells were finally scanned with the fluorescence microplate reader (Molecular Devices, Inc., Sunnyvale, California, USA) and the fluorescence was analyzed. Red emission of the dye represented a potential-dependent aggregation in the mitochondria, reflecting MMP. Green fluorescence represented the monomeric form of JC-1, appearing in the cytosol after mitochondrial membrane depolarization.

### 2.8. Intracellular Cyto-C Measurement

AML-12 cells were cultured in a 96-well plate at a density of 5,000 cells/well and treated with 0.5 mM H_2_O_2_ for 20 min. Then H_2_O_2_ was discarded and the cells were incubated with Salvianolate at the concentration of 6.25 *μ*g/mL, 12.5 *μ*g/mL, and 25 *μ*g/mL for a period of 24 hours. After 24 hours, cells were washed with cold PBS twice and fixed in 4% formaldehyde. After washing by PBS, the cells were then blocked with 5% bovine serum albumin in PBS buffer for 30 min at room temperature before being incubated with the Cyto-C (1 : 400) primary antibodies (Beyotime, Haimen, China). Cells were then stained with FITC-conjugated secondary antibodies. After washing, cells were double-stained with Hoechst 33342 (Beyotime, Haimen, China) to visualize the nuclei. Images were taken by Cellomics ArrayScan VTI HCS Reader.

### 2.9. Animals and Experimental Design

For the experiment, 43 heads of specific pathogen-free male Wistar rats weighing 150 ± 10 g were obtained from Shanghai Laboratory Animal Center, Chinese Academy of Science (Shanghai, China), and acclimated for at least 1 week before the experiment. All animals were housed in an environmentally controlled room at 22 ± 2°C and 60 ± 5% relative humidity under 12/12 h light/dark cycle. All rats were housed under controlled temperature (22°C), humidity (55%), and lighting (12-hour artificial light and dark cycle), with free access to tap water and rat chow. The standard diet pellets contained not less than 20% protein, 5% fibers, 3.5% fats, and 6.5% ash and vitamins mixture. To establish hepatocyte injury model* in vivo*, 43 male Wistar rats were initially divided into two groups as the normal control group (*n* = 10, olive oil injection) and model group (*n* = 33, CCl_4_ injection). Firstly, rats in model group received 100% CCl_4_ at dose of 3 mL·kg^−1^ with a single subcutaneous injection. 6 h later, the model rats were further divided into 3 groups as follows: the model control group (*n* = 11, distilled water and CCl_4_ injection), Salvianolate group (*n* = 11, 40 mg·kg^−1^ of Salvianolate and CCl_4_ injection), and NAC group (*n* = 11, 500 mg·kg^−1^ of NAC and CCl_4_ injection). Then, except for the rats in normal control group, which received only pure olive oil, rats in other groups were injected with 50% CCl_4_-olive oil solution at dose of 2 mL·kg^−1^ subcutaneously once a week for 4 times. Subsequently, rats in Salvianolate group were injected intraperitoneally with Salvianolate at a daily dose of 40 mg·kg^−1^ for 2 weeks. Rats in NAC group were treated with NAC orally at the dose of 500 mg·kg^−1^ in the same period of time. All rats were starved for 12 hours after the last treatment, and then they were sacrificed. The serum and liver samples were harvested under pentobarbital sodium anesthesia.

Our experiments conformed to the ethical guidelines outlined in the Guide for the Care and Use of Laboratory Animals by the Laboratory Animal Center, Shanghai University of Traditional Chinese Medicine.

### 2.10. Measurements of Serum Indicators of Liver Function

Activities of serum alanine transaminase (ALT), aspartate aminotransferase (AST), and total bile acid (TBA) were quantitated by using commercial kits following the instructions provided by the manufacturer (Nanjing Jian Cheng Bioengineering Institute, Nanjing, China), including the use of standardization.

### 2.11. Parameters for Peroxidative Damage in Liver Tissue

Hepatic homogenates were centrifuged at 3,000 rpm for 20 min at 4°C. Supernatants were immediately collected and assayed for enzyme activities. Levels of malondialdehyde (MDA), glutathione (GSH), and antisuperoxideanion free radical (ASAFR) were assayed according to the protocols of kits purchased from Nanjing Jian Cheng Bioengineering Institute. All these parameters were expressed by gram protein which was determined by the BCA protein assay kit (Pierce, Thermo Scientific, Rockford, USA) according to the manufacturer's protocol using bovine serum albumin as a standard.

### 2.12. Histopathological and Immunohistological Analysis of Liver Tissue

Liver tissues were fixed in buffered formalin and embedded in paraffin wax. After routine processing, liver sections of 4 *μ*m thickness underwent staining with H&E. Semiquantitative analysis of liver injury was performed according to the nonalcoholic fatty liver disease activity score (NAS): (1) hepatic steatosis: 0 (<5%); 1 (5%–33%); 2 (34%–66%); and 3 (>66%) and (2) ballooning degeneration of liver cell: 0, none; 1, rare; and 2, many [12]. Immunohistological analyses for Cyto-C and 4-hydroxynonenal (4-HNE) were performed by using anti-Cyto-C and anti-4-HNE antibodies (Abcam, ab46545) and visualized by ChemMate*™* Envision*™* Detection Kit (Dako). The section was pretreated using heat mediated antigen retrieval with sodium citrate buffer (pH 6, epitope retrieval solution 1) for 20 mins. The section was then incubated with the first antibody, 1 *μ*g/mL, for 15 minutes at room temperature and detected using HRP conjugated compact polymer system. DAB was used as the chromogen. The section was then counterstained with hematoxylin. All the images were analyzed with a light microscope (Olympus BX40, Japan).

### 2.13. Liver Perfusion and Processing for Ultrastructural Analysis

For ultrastructural analysis, the rat liver was cleared by perfusion with PBS of room temperature through the portal vein at a flow rate of 3 mL·min^−1^. One minute later, 2.5% glutaraldehyde was perfused for an additional one minute at the same flow rate. Whereafter, livers were quickly removed and thoroughly immersed in 2.5% glutaraldehyde for 48 hours at 4°C. Subsequently, the liver tissues were cut from the fixative for TEM, respectively, as described previously [13].

### 2.14. Statistical Analysis

All data were analyzed by using PASW Statistics 18 software. For measurement data, differences between the groups were assessed by nonparametric one-way analysis of variance. Values in the text are presented as mean ± SD. For enumeration data, the results were performed by ridit-analysis. *p* < 0.05 was considered statistically significant.

## 3. Results

### 3.1. Salvianolate Inhibited H_2_O_2_-Induced Hepatocyte Injury* In Vitro*

**Figure 1:**
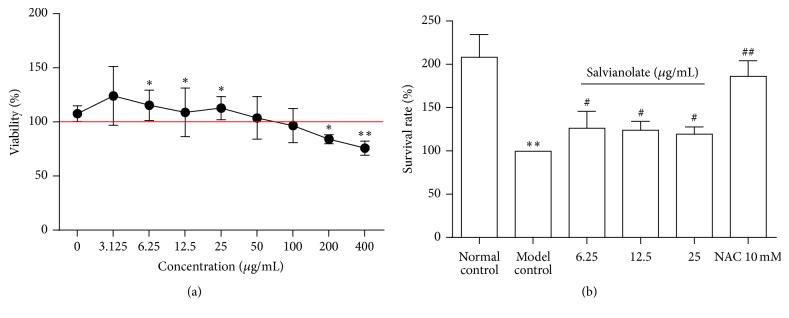
Cytotoxicity and proliferation of Salvianolate in AML-12 cell lines. (a) AML-12 cells were cultured in a 96-well plate at a density of 5,000 cells/well and incubated with Salvianolate with concentrations of 3.125–400 *μ*g/mL for 24 h. (b) AML-12 cells were cultured in a 96-well plate at a density of 5,000 cells/well and exposed to 0.5 mM H_2_O_2_ for 20 min; then the cells were incubated with Salvianolate with concentrations of 6.25–25 *μ*g/mL or NAC with concentration of 10 mM for 24 h. Both cytotoxicity of Salvianolate and proliferation of Salvianolate or NAC were detected by CCK8. ^*∗*^ *p* < 0.05 and ^*∗∗*^ *p* < 0.01 versus normal control; ^#^ *p* < 0.05 and ^##^ *p* < 0.01 versus model control.

H_2_O_2_ was widely used to induce hepatocyte injury for the classical oxidative stress injury model* in vitro* [14, 15]. Our previous study found that the proper working concentration of H_2_O_2_ was 0.5 mM, which caused cell viability decrease by about 40% in 30 minutes [16]. Therefore, we exposed AML-12 cells to concentration of 0.5 mM H_2_O_2_ for 30 minutes to establish an oxidative stress injury model in this study. To evaluate the most effective concentration of Salvianolate, H_2_O_2_-induced AML-12 cells were treated with different concentrations of Salvianolate from 3.125 to 400 *μ*g/mL progressively. We found that the survival rate was 100% in the normal control group and Salvianolate presented the nontoxic concentration from 3.125 to 100 *μ*g/mL (Figure 1(a)). To examine the cytoprotective effects of Salvianolate on oxidative stress-induced hepatocyte injury, AML-12 cells were exposed to 0.5 mM H_2_O_2_ for 20 min to induce hepatocyte injury. Subsequently, cells were incubated with 3.125–12.5 *μ*g/mL Salvianolate or 10 mM NAC as positive drug for 24 h. Then the cell viability was detected by the CCK8 assay. As shown in Figure 1(b), 0.5 mM H_2_O_2_ induced a significant level of cell damage (*p* < 0.05). In contrast, both Salvianolate and NAC effectively protected against hepatocyte injury induced by H_2_O_2_ *in vitro* (*p* < 0.05), and NAC had a stronger protective effect than Salvianolate (*p* < 0.05) (Figure 1(b)).

### 3.2. Salvianolate Ameliorated H_2_O_2_-Induced Mitochondrial Injury in Hepatocytes* In Vitro*

**Figure 2:**
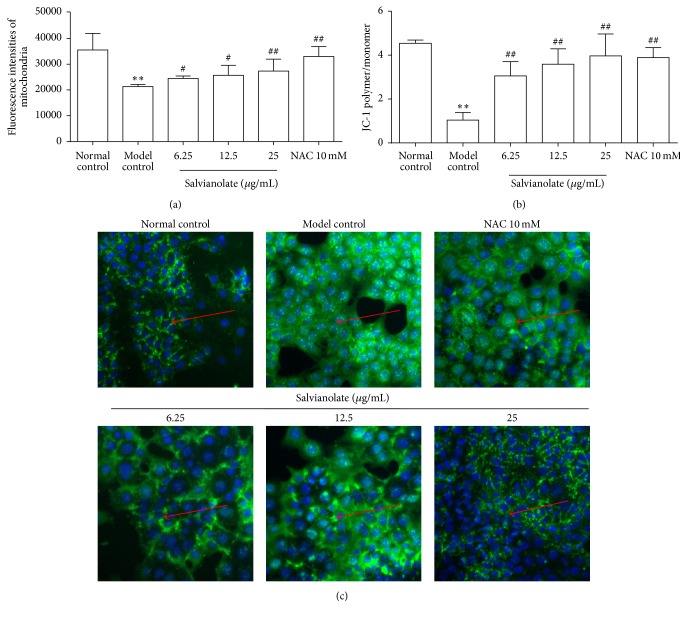
Protective effects of Salvianolate on H_2_O_2_-induced mitochondrial injury in AML-12 cells* in vitro*. Hepatocytes injury model was established with 0.5 mM H_2_O_2_, and then the cells were incubated with Salvianolate with concentrations of 6.25, 12.5, or 25 *μ*g/mL or NAC with concentration of 10 mM for 24 h. (a) Semiquantification data for expressions of viable mitochondria in AML-12 cells by quantifying the fluorescence intensity of mitochondria probe with MitoTracker Green kits. (b) Semiquantification data for expression of MMP in AML-12 cells by examining the fluorescence intensity ratio of JC-1 aggregation/JC-1 monomer. (c) Expression of Cyto-C (green fluorescence marked with red arrows) in cytoplasm was revealed by the images taken by Cellomics ArrayScan VTI HCS Reader. ^*∗∗*^ *p* < 0.01 versus normal control; ^#^ *p* < 0.05 and ^##^ *p* < 0.01 versus model control.

Based on the results above, we found that Salvianolate could protect AML-12 cells from H_2_O_2_-induced injury at the concentrations of 6.25 *μ*g/mL, 12.5 *μ*g/mL, and 25 *μ*g/mL. To confirm whether Salvianolate ameliorated hepatocytes by protecting mitochondria against injury, we further evaluated the effects of treatment with Salvianolate for 24 h on expressions of viable mitochondria, MMP dissipation, and expression of Cyto-C in cytoplasm following the exposure to 0.5 mM H_2_O_2_ for 20 min* in vitro*. Firstly, we quantified the viable mitochondria expression upon H_2_O_2_-induced oxidative stress with or without Salvianolate by quantifying the fluorescence intensity of mitochondria probe with MitoTracker Green kits. As shown in Figure 2(a), the fluorescence intensity of mitochondria probe in model control group was obviously weakened compared with that in normal control group. Notably, mitochondria probe fluorescence intensity was enhanced by incubation with NAC and dose-dependent Salvianolate. Simultaneously, we examined the fluorescence intensity ratio of JC-1 aggregation/JC-1 monomer. As the results revealed in Figure 2(b), the ratio of JC-1 aggregation/JC-1 monomer was sharply decreased in the model control group, while treatment of Salvianolate or NAC significantly attenuated the condition. To further confirm the protective effect of Salvianolate on hepatocyte mitochondria, we investigated the effect of Salvianolate on the expression of Cyto-C in AML-12 cells cytoplasm. As shown in Figure 2(c), expression of Cyto-C in cytoplasm was higher in the model control group compared with that in the normal control group, and treatment with Salvianolate significantly attenuated the levels of Cyto-C.

### 3.3. Salvianolate Attenuated CCl_4_-Induced Hepatic Inflammation in Rats

**Figure 3:**
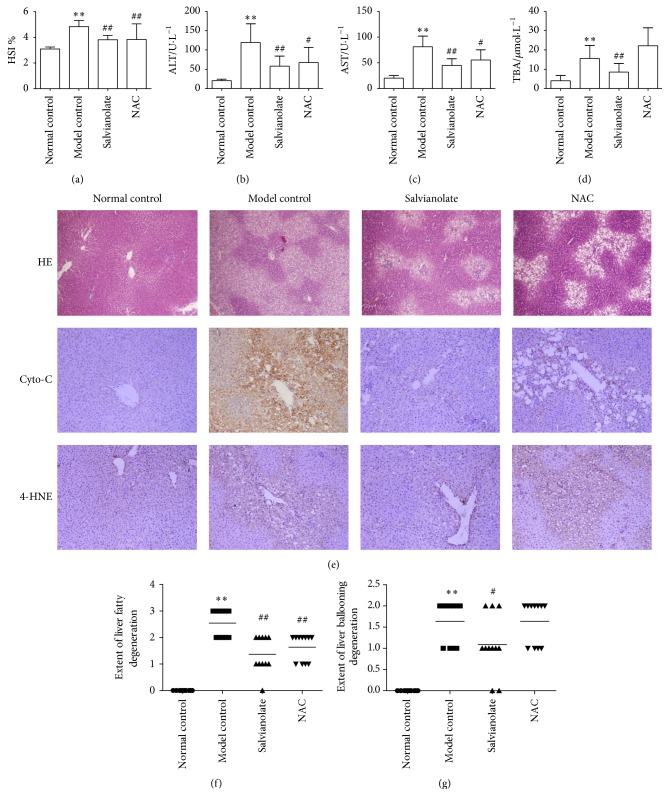
Protective effects of Salvianolate on CCl_4_-induced liver injury in rats. Male Wistar rats were treated as described in the legend. Serum ALT (b), AST (c), and TBA (d) were assessed by liver function tests kits, respectively. Levels of serum liver functions were decreased, and HSI (a) was alleviated by Salvianolate treatment with dose of 40 mg/kg in CCl_4_-induced liver injury mice. (e) Histologic evaluation of liver tissues was stained with H&E, and the expression of Cyto-C and 4-HNE was assayed by IHC (×200). Semiquantitative analysis of liver injury was performed according to the nonalcoholic fatty liver disease activity score (NAS): (f) hepatic steatosis: 0 (<5%); 1 (5%–33%); 2 (34%–66%); and 3 (>66%) and (g) ballooning degeneration of liver cell: 0, none; 1, rare; and 2, many. ^*∗∗*^ *p* < 0.001, versus normal control; ^#^ *p* < 0.05 and ^##^ *p* < 0.001, versus model control.

Next, we investigated the effects of Salvianolate on CCl_4_-induced liver injury to further confirm the role of Salvianolate in protecting hepatocyte mitochondria injury* in vivo*. As shown in Figures 3(a)–3(d), hepatopancreas somatic indices (HSI) and levels of serum indicators of liver function such as ALT, AST, and TBA were all upregulated after CCl_4_ administration. Salvianolate, as well as NAC, could significantly downregulate the serum liver functions. Furthermore, we investigated the effects of Salvianolate on liver inflammation by H&E staining. As shown in Figure 3(e), intact lobe structure with tidy hepatic cords and no degeneration, necrosis, or inflammatory cells infiltration were observed in normal control group. Meanwhile, a considerable mass of fat vacuoles in hepatic tissue accompanied with mononuclear cells infiltration alongside the portal area and central vein was shown in model group, as well as ballooning and hydropic degeneration of hepatocytes. After treated with Salvianolate or NAC, those pathological changes were alleviated notably. Additionally, ridit-analysis showed that treatment with CCl_4_ led to fatty and ballooning degeneration significantly, while those pathological changes in Salvianolate 40 mg/kg group were strikingly reversed. However, the alleviative effect of NAC was observed only on inhibiting hepatocyte fatty degeneration (Figure 3).

### 3.4. Salvianolate Alleviated CCl_4_-Induced Liver Oxidative Stress Injury in Rats

**Figure 4:**
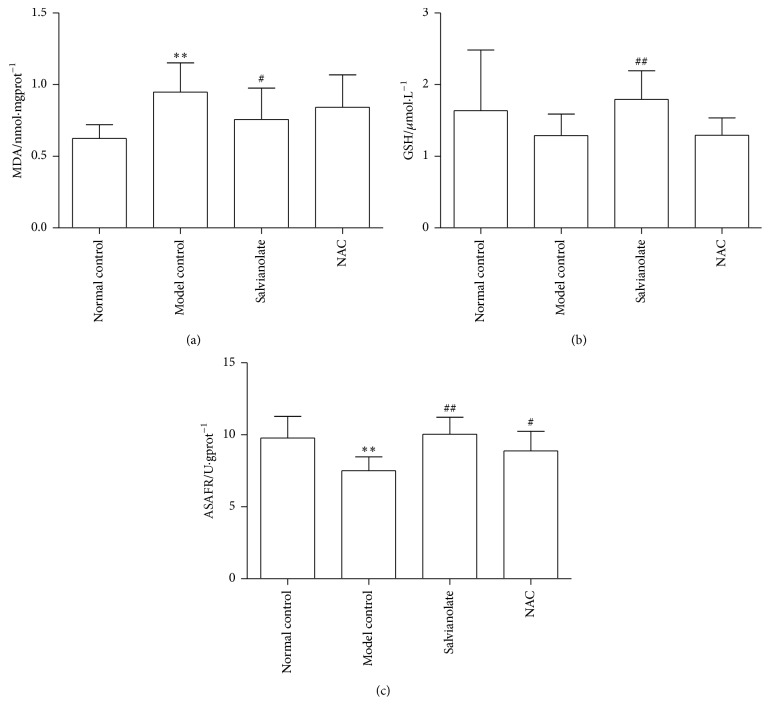
Effects of Salvianolate on CCl_4_-induced liver oxidative stress injury in rats. Levels of parameters for peroxidative damage MDA, GSH, and ASAFR in hepatic homogenates were assayed according to the protocols of corresponding kits. ^*∗∗*^ *p* < 0.01 versus normal control; ^#^ *p* < 0.05 and ^##^ *p* < 0.01 versus model control.

Besides, tissue oxidative stress parameters including MDA, GSH, and ASAFR were also tested to observe the effect of Salvianolate on liver oxidative stress injury. As shown in Figures 4(a)–4(c) and 3(e), after injury by CCl_4_, higher levels of MDA and 4-HNE and lower GSH and ASAFR were presented in the model control group compared with those in the normal control group. Treatment of Salvianolate could downregulate the level of MDA and upregulate the levels of GSH, ASAFR, and 4-HNE (Figures 4(a), 4(b), and 3(e)).

### 3.5. Salvianolate Relieved Hepatocyte Mitochondrial Injury* In Vivo*

**Figure 5:**
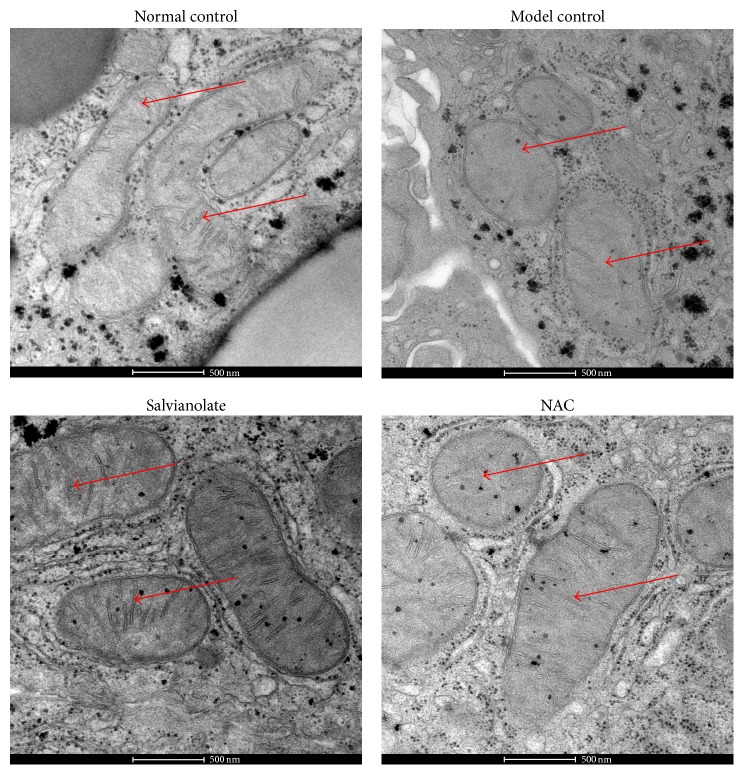
Salvianolate relieved mitochondrial injury in hepatocytes. Normal hepatocytes and mitochondria were observed in normal group. However, after stimulating with CCl_4_, cellular nucleus concentrating, mitochondria swelling and mitochondria cristae fragmentation or disappearance, and so forth were commonly seen in model control (marked with red arrows). Meanwhile, all those pathological changes were ameliorated after the rats were given 40 mg/kg Salvianolate or NAC. Images of TEM for mitochondria in hepatocytes ×13800.

To further evaluate the effect of Salvianolate on CCl_4_-induced hepatocyte injury* in vivo*, the morphology of liver mitochondria was observed by TEM. As shown in Figure 5, normal hepatocytes were observed in normal group and mitochondrial configuration differed with sphericity, rod or filament. Besides, mitochondrial cristae, resulting from inner mitochondrial membrane (IMM) sinking into matrix, were arranged closely. However, after stimulating with CCl_4_, pathological characteristics of hepatocytes such as cellular nucleus concentrating, mitochondria swelling, and mitochondria cristae fragmentation or disappearance were commonly detected. However, all those pathological changes were ameliorated after the rats were given Salvianolate or NAC.

## 4. Discussion

Oxidative stress is an important determinant in the pathophysiology of many hepatic diseases, for example, alcoholic hepatitis, viral hepatitis, liver fibrosis, and even liver cancer [17]. A growing body of evidences suggest that oxidative stress due to excessive production of ROS is associated with hepatocyte mitochondria injury [18]. Mitochondria are important organelles in eukaryotic cells, playing a significant role in cellular energy production. Once mitochondria are damaged by excessive ROS, the process of cell apoptosis and death can be mediated and accelerated [19]. Therefore, attempts to target ROS have been based on increasing antioxidant bioavailability and reducing ROS generation. Because of their cytotoxicity, CCl_4_ and H_2_O_2_ have been widely used in various experiments* in vivo* or* vitro* with their activities to induce oxidative stress, resulting in the imbalance between oxidation and antioxidation [20, 21]. Thus, we employed H_2_O_2_ and CCl_4_ in our study to investigate the effect of Salvianolate on hepatocytes oxidative stress injury* in vitro* and* in vivo*, respectively.

*Salvia miltiorrhiza*, a traditional Chinese medicine, has been used as a common clinical medicine for thousands of years. Salvianolate, as the main water soluble component of* Salvia miltiorrhiza*, has been proved to exert strong protective effects on vascular disorders with its antioxidant function [11]. However, the effects of Salvianolate on liver diseases via protecting mitochondria from oxidative stress remain not entirely clear yet.

In order to explore the effect of Salvianolate against liver injury, we investigated the role of Salvianolate in hepatocyte injury* in vitro* and* in vivo*, respectively. Firstly, toxicological and pharmacological studies on Salvianolate were performed on mouse hepatocytes (AML-12 cell line)* in vitro*, which implied that the Salvianolate could inhibit H_2_O_2_-induced hepatocyte injury at multiple concentrations without obvious cytotoxicity on hepatocytes. Secondly, we investigated if Salvianolate could protect the hepatocytes by inhibiting mitochondria injury. As is known to us, it is important for mitochondria to maintain physiological function with normal MMP, while MMP dissipation is considered to be a common early event of apoptosis [22]. Transformation of JC-1 fluorescence that turns from red to green indicates the descent of MMP, so it can be adopted to determine whether the cells are in the early apoptosis stage. The JC-1 staining showed that hepatocytes MMP decreased significantly after stimulating by H_2_O_2_, while treatment with Salvianolate for 24 h could increase MMP remarkably in a dose-dependent manner. Cyto-C is an initiation factor of cell apoptosis locating in intermembrane space between inner and outer mitochondrial membrane. When hepatocytes are stimulated by excessive ROS, permeability transition of mitochondria changes and Cyto-C will be released into plasma from mitochondria. Our study revealed that Salvianolate could decrease the expression of Cyto-C and 4-HNE in damaged hepatocyte plasma significantly compared with model group, which indicated that Salvianolate could keep mitochondrial membrane integrality via antagonizing H_2_O_2_-induced oxidative stress.

Finally, to confirm the effect of Salvianolate* in vivo*, we investigated the effect of Salvianolate on hepatocyte mitochondria in CCl_4_-induced liver injury model with rats. Salvianolate could alleviate levels of serum liver function in CCl_4_-induced liver injury rats. It was obvious that Salvianolate could relieve the infiltration of inflammatory cells and degeneration of hepatocytes. Besides, level of the indicator of membrane peroxidation, MDA, was decreased, while the main nonenzymatic-ROS scavengers in liver cells such as GSH and ASAFR were increased obviously by Salvianolate treatment. However, the results of serum liver function and histopathological assessment indicated that the antioxidant effect of Salvianolate was observed only when it was used at dose of 40 mg/kg. These results suggested that Salvianolate had potential anti-injury effect on CCl_4_-induced liver injury.

## 5. Conclusions

Above all, Salvianolate is effective in alleviating hepatocytes injury via protecting mitochondria* in vitro* and* in vivo*, which might provide alternative treatment options for hepatic injury.

## Abbreviations

ALT: Alanine transaminase
AML-12: Alpha mouse liver 12
ASAFR: Antisuperoxideanion free radical
AST: Aspartate aminotransferase
ATP: Alanine aminotransferase
CCCP: Carbonyl cyanide m-chlorophenylhydrazone
CCK: Cell Counting Kit
CCl_4_: Carbon tetrachloride
Cyto-C: Cytochrome C
FBS: Fetal bovine serum
GSH: Glutathione
H&E: Hematoxylin and eosin
HIS: Hepatopancreas somatic indices
4-HNE: 4-Hydroxynonenal
H_2_O_2_: Hydrogen peroxide
IHC: Immunological Histological Chemistry
JC-1: 5,5′,6,6′-Tetrachloro-1,1′,3,3′-tetraethylbenzimidazole-carbocyanide iodine
MDA: Malondialdehyde
MMP: Mitochondrial membrane potential
NAC: N-Acetyl-L-cysteine
NAS: Nonalcoholic fatty liver disease activity score
PBS: Phosphate-buffered saline
ROS: Reactive oxygen species
TBA: Total bile acid
TEM: Transmission electron microscope.
